# Microarray Analysis Confirms ImmunoCAP-Fluorescence Enzyme Immunoassay Results on Specific IgE in Patients with Atopic Dermatitis and Suspected Birch Pollen-Related Food Allergy

**DOI:** 10.1159/000522525

**Published:** 2022-04-04

**Authors:** Anja Wassmann-Otto, Annice Heratizadeh, Katja Wichmann, Thomas Werfel

**Affiliations:** Division of Immunodermatology and Allergy Research, Department of Dermatology and Allergy, Hannover Medical School, Hannover, Germany

**Keywords:** Allergen microarray ImmunoCAP® ISAC, Atopic dermatitis, Birch pollen-related food allergy, Food challenge, Late eczematous reactions

## Abstract

**Background:**

Previous studies demonstrated that birch pollen-related foods can cause late eczematous responses in birch pollen-sensitized patients with atopic dermatitis (AD). However, suitable markers to predict birch pollen-related food allergy in patients with AD are still lacking.

**Objective:**

We evaluated the correlation of the results from ImmunoCAP® fluorescence enzyme immunoassay (FEIA) singleplex and ImmunoCAP® immuno solid-phase allergen chip (ISAC) multiplex system in AD patients and investigated the diagnostic validity of allergen microarray analysis, measuring specific IgE (sIgE) with ImmunoCAP® ISAC to predict birch pollen-related food allergy in patients with AD.

**Methods:**

A total of 19 children and adults with AD, existing IgE-mediated birch pollen sensitization, and suspected birch pollen-related food allergy underwent a double-blind placebo-controlled food challenge (DBPCFC) in the clinical routine. Total and sIgE levels to birch pollen, Bet v 1, Bet v 2, and birch pollen-related foods (apple, carrot, celery, and hazelnut) were determined prior to the DBPCFC by ImmunoCAP®-FEIA. Additionally, allergen microarray ImmunoCAP® ISAC analysis was performed. Data were analyzed retrospectively.

**Results:**

Twelve out of 19 patients (63% responders) experienced an allergic reaction upon DBPCFC. Overall, 7 patients (37%) developed a significant deterioration of AD with a median increase of 12.4 points in the scoring of atopic dermatitis (SCORAD) index (range 10.0–15.7). Oral allergy syndrome was the predominant immediate-type symptom (*n* = 11/12 responders). There were no differences in sensitization frequencies regarding allergens of the pathogenesis-related protein family 10 between responders and non-responders. In all patients, correlation of IgE levels determined with ImmunoCAP® ISAC and ImmunoCAP®-FEIA, respectively, was significant with high correlation coefficients regarding birch pollen allergen extract, rBet v 1, and rBet v 2 (*r*<sub>s</sub> > 0.8, *p* < 0.001) and lower but also significant correlation coefficients regarding food allergens (*r*<sub>s</sub> < 0.8, *p* < 0.05–<0.001).

**Conclusion:**

ImmunoCAP® ISAC microarray allows displaying a differentiated sensitization profile in birch pollen-sensitized patients with AD. However, IgE-mediated sensitization against birch pollen-related allergens revealed by the allergen multiplex system does not predict late eczematous reactions upon DBPCFC with birch pollen-related foods.

## Introduction

In central Europe, birch pollen-related food antigens are the most frequent elicitors of pollen-related food allergies [1]. The majority of immunological cross-reactions between pollen and corresponding foods can be contributed to the major birch pollen allergen Bet v 1, which belongs to the pathogenesis-related protein family 10 (PR-10) [2]. Over the last decades, a multitude of PR-10-homolog allergens have been characterized in plant foods across different botanical families [2, 3, 4, 5, 6, 7, 8, 9, 10, 11, 12]. Birch pollen-related food allergens typically induce immediate-type reactions with a wide spectrum of clinical reactions ranging from most frequently observed oral allergy syndrome (OAS) up to anaphylactic reactions which are reported in single cases [9, 10, 11, 13, 14, 15]. Furthermore, there is evidence for late-type eczematous reactions to birch pollen-related foods in patients with atopic dermatitis (AD): in a cohort of 37 adults [16] and in a subsequent study with 12 children [17], we previously demonstrated for the first time that birch pollen-related foods are able to cause a worsening of eczema even in the absence of immediate-type reactions. Recently, we could confirm these observations in a larger cohort including 182 children and adult patients with AD who underwent double-blind placebo-controlled food challenges (DBPCFC) with birch pollen-related foods. Here, a worsening of AD was observed in 18% of the patients [18].

So far, patients experiencing late-type eczematous reactions to birch pollen-related food cannot be identified by a distinct sensitization profile. In the last decade, protein microarrays have been introduced as a new diagnostic tool into research and diagnostic workup [19, 20, 21, 22, 23, 24, 25, 26, 27, 28, 29, 30, 31, 32]. Using purified recombinant and native allergen components microarray assays allow allergy diagnostics on a molecular level. According to the WAO/ARIA/GA2LEN consensus document on molecular-based allergy diagnostics, allergen microarrays can be considered as a third-line approach along with clinical case history (first-line) and allergen extract-based IgE tests (second-line) to be used in inconclusive cases [32]. Since 2001, the immuno solid-phase allergen chip (ISAC) system is available [33]. Although reliability and diagnostic validity of this microarray has been the subject of intense research observing cohorts of atopic patients [19, 22, 24, 25, 26, 27, 30, 34], only limited data are available for the diagnostic use of ImmunoCAP® ISAC in AD [20, 23, 29, 35]. In particular, clinical implications of ImmunoCAP® ISAC in diagnosing birch pollen-related food allergy in children and adult patients with AD have not been explored so far.

Thus, in the present study, we investigated the diagnostic validity of ImmunoCAP® ISAC for the diagnosis of immediate- and/or late-type reactions to birch pollen-related food in children and adults with AD. We aimed to better define sensitization profiles in these patients and, moreover, to evaluate whether ImmunoCAP® ISAC test results are valid predictors to identify patients experiencing a late eczematous response upon ingestion of birch pollen-related foods. Furthermore, we intended to correlate allergen microarray test results to that of an established fluorescence enzyme immunoassay (FEIA).

## Methods

### Patients

A total of 19 highly selected patients with physician-diagnosed mild to severe AD according to the criteria of Hanifin and Rajka [36] and suspicion of birch pollen allergy with an IgE-mediated sensitization to birch pollen of at least ≥3.5 kU/L were included in the retrospective study. All patients were suspected to exhibit immediate-type and/or late-type allergy to at least one birch pollen-related food (apple, carrot, celery, or hazelnut) based on the patient's history. Therefore, all patients already had consecutively undergone oral food challenges with birch pollen-related foods as a routine diagnostic procedure in the Department of Dermatology and Allergy, Hannover Medical School, Germany according to the guidelines on food allergy [37, 38]. Prior to oral food challenges, written informed consent had been obtained from all patients. As mentioned above, corresponding data were analyzed retrospectively.

The included study population consists of a subcohort from our previous publication which comprised a cohort of 182 patients with AD [18]. The microarray test results presented and discussed in this paper have not yet been published.

#### Medical History and Scoring of AD

A detailed medical history of AD and respiratory and birch pollen-related food allergy was obtained. Severity of AD was determined according to the to the “scoring atopic dermatitis” (SCORAD) index [39, 40], combining clinical signs and subjective symptoms of AD. As stated in the recommendations of the European Academy of Allergology and Clinical Immunology (EAACI) and the Global Allergy and Asthma European Network (GA2LEN) regarding eczematous reactions to food in patients with AD [41], the severity of AD was rated daily during DBPCFC until 1 day of follow-up.

#### Total and Specific IgE

Prior to DBPCFC, total IgE and specific IgE (sIgE) titers against birch pollen, rBet v 1, and rBet v 2 as well as selected birch pollen-related foods (apple, carrot, celery, and hazelnut) had been determined using the ImmunoCAP® fluorescence enzyme immunoassay (FEIA) according to the manufacturers' instructions (Thermo Fisher Scientific Inc., Waltham, MA, USA; manufactured: Phadia AB, Uppsala, Sweden). Birch pollen- or food-specific IgE titers of at least 0.35 kU/L were defined as sensitization. Since the data were analyzed retrospectively, sIgE titers to recombinant birch pollen allergens, rBet v 1, and rBet v 2 were not available for all patients (*n* = 13/19 and *n* = 8/19, respectively).

#### Allergen Microarray Analysis

Additionally, patient's sera were analyzed by using a semiquantitative microarray system (ISAC-103, ImmunoCAP®) that included a total of 103 native and recombinant allergen components according to the manufacturers' instructions (Thermo Fisher Scientific Inc.). For the present study, exclusively, allergens of plant origin were included in the analysis (58 allergen components: 31 aeroallergens and 27 food allergens) with the main focus on birch pollen allergens as well as aeroallergens and food allergens of the PR-10 allergen family. Other aero- and food allergens of plant origin are displayed in the supplement. The lower limit of detection of this microarray is 0.3 ISAC standardized units (ISUs). Hence, test results showing an IgE antibody titer ≥0.3 ISU were regarded as positive, 0.3–0.9 ISU as low, 1.0–14.9 ISU as moderate/high, and antibody titers ≥15.0 ISU were regarded as very high [25].

#### Double-Blind Placebo-Controlled Food Challenges

After a 4-week elimination diet upon instruction of a dietician, AD patients underwent consecutively DBPCFC with at least one birch pollen-related food (apple, carrot, celery, or hazelnut) in clincal routine. DBPCFC procedures followed national and international guidelines [42, 43]. All DBPCFC were performed outside the birch pollen season and under stable skin conditions. If required, ongoing topical therapy with mild corticosteroids (European class I or II) was allowed but had to remain steady until follow-up day 1. The intake of antihistamines had to be stopped 72 h prior to the DBPCFC.

DBPCFC has been performed according to the standard procedure as previously published [16]. Verum and the placebo were identical in color, consistency, and taste and were masked with orange flavor (Nutricia, Erlangen, Germany) and beta-carotene (Synopharm, Barsbüttel, Germany). Verum meals containing two to four different birch pollen-related foods were prepared with 50 g of fresh apple (Granny Smith), 50 g of fresh carrot, 50 g of fresh celery, and/or 10 g of hazelnut and were masked with carob (Sinlac®, Nestlé, Frankfurt, Germany) and rice flakes (Demeter Bauckhof, Rosche, Germany). For oral food challenges with only one birch pollen-related food, the amount of food in the verum meal was adapted to a normal daily intake and contained 150 g apple, 150 g carrot, 150 g celery, and 10 g hazelnut, respectively. The placebo consisted of carob, rice flakes, orange flavor, and beta-carotene.

DBPCFC was performed in a two-step procedure built up of a “spit-and a swallow phase” as firstly described by Ballmer-Weber et al. [13]. On the first day of testing a new meal, the patients had to keep 15 g of it in their mouth and spit it out after 10 and 30 s, respectively. Thereafter, the “swallow phase” was performed: verum/placebo was administered and titrated with increasing doses at every 30 min (15, 45, 90, and 180 g) until an allergic reaction occurred or the full dose was given. In case no immediate-type reaction had occurred on day one, the complete dose was administered at once on the second day. Patients were observed up to 3 h after the last dose had been given. AD severity was rated by a physician every morning during the challenge until follow-up using the SCORAD index.

Clinical reactions that occurred within 6 h after ingesting the last dose were defined as immediate-type reactions. In accordance to the definition of the EAACI and GA2LEN position paper [41], an increase of ≥10 SCORAD points occurring within 6–48 h after administration of the last dose was considered as a significant deterioration of AD (late eczematous reaction). Combined reactions were defined as an immediate-type reaction prior to a worsening of AD.

#### Statistical Analysis

As the data showed a nonnormal distribution, the statistical analysis was performed by nonparametric tests. The medians of metrically scaled variables (age, total and sIgE titers, and SCORAD) were compared with the Mann-Whitney U-test. The history of respiratory atopic diseases of responders and non-responders was compared with the Fisher exact test. The bivariate correlation between test results measured with ImmunoCAP® singleplex and ImmunoCAP® ISAC multiplex system was calculated with Spearman's rank correlation coefficient (nominal scale: positive detection of IgE antibodies/negative detection of IgE antibodies).

## Results

### Demographics and Baseline Characteristics

A total of 19 patients (58% female and 42% male) were included in the study. The median age of the study population was 36 years (range 5–70 years). The median disease severity of AD assessed by the SCORAD index was 26.6 points (range 11.4–51.8 points). Five out of 19 patients suffered from seasonal allergic rhinoconjunctivitis (RCA), 1 patient suffered from allergic asthma bronchiale, and 4 patients had both seasonal RCA and allergic asthma bronchiale.

#### Worsening of AD upon Food Challenge in More than One-Third of the Subjects

Twelve out of 19 patients (63% responders) experienced any allergic reaction upon DBPCFC with birch pollen-related foods: 5 responders reacted with an isolated immediate-type reaction, 6 patients developed a combined immediate- and late-type reaction with an eczematous response, and 1 patient developed an isolated late-type reaction with worsening of AD. Hence, a total of 7 out of 19 patients (37%) showed a worsening of AD after ingestion of birch pollen-related foods (median SCORAD increase 12.4 points, range 10.0–15.7 points).

OAS was the predominant immediate-type symptom (*n* = 11). Only 1 patient developed an immediate-type reaction with gastrointestinal symptoms (diarrhea) in addition to an OAS upon ingestion of a meal containing the four birch pollen-related foods.

Seven patients did not react to the tested birch pollen-related foods in DBPCFC in this study (37% non-responders). No clinical reactions have been observed upon placebo challenges.

#### No Difference in AD Frequency of Inhalant Allergies, Age, and Severity between Responders and Non-Responders

Responders showed a clear trend for a higher prevalence of RCA compared to non-responders (67% vs. 14%, respectively). However, this difference did finally not reach the level of statistical significance due to small sample size (*p* > 0.50, Fisher exact test). No difference between both study groups could be observed in terms of frequency of asthma bronchiale, age, or disease severity of AD based on the SCORAD index (data not shown).

#### ImmunoCAP®-FEIA Showed No Significant Differences Regarding sIgE Titers between Responders and Non-Responders

The study population was characterized by sensitization to birch pollen determined by serum sIgE with a median value of 47.70 kU/L (range 4.06–>100.00 kU/L) detected by ImmunoCAP®-FEIA. Bet v 1-specific IgE could be detected in all responders and non-responders (available data: *n* = 13 responders and non-responders), whereas no patient was sensitized to birch pollen profilin Bet v 2 (available data: *n* = 8 responders and non-responders).

The median sIgE titers to birch pollen allergens and birch pollen-related foods measured by ImmunoCAP®-FEIA in sera of responders and non-responders are summarized in Table 1. No significant differences regarding sIgE titers could be observed between responders und non-responders.

#### Investigation of a Broader Spectrum of Sensitizations to Birch Pollen-Related Allergens in AD Patients by ImmunoCAP® ISAC

In the following, ImmunoCAP® ISAC test results from birch pollen allergens, PR-10-related aeroallergens, and PR-10-related food allergens are presented. Background sensitizations analyzed by ImmunoCAP® ISAC microarray are displayed in the online supplementary material (see www.karger.com/doi/10.1159/000522525 for all online suppl. material).

**Fig. 1:**
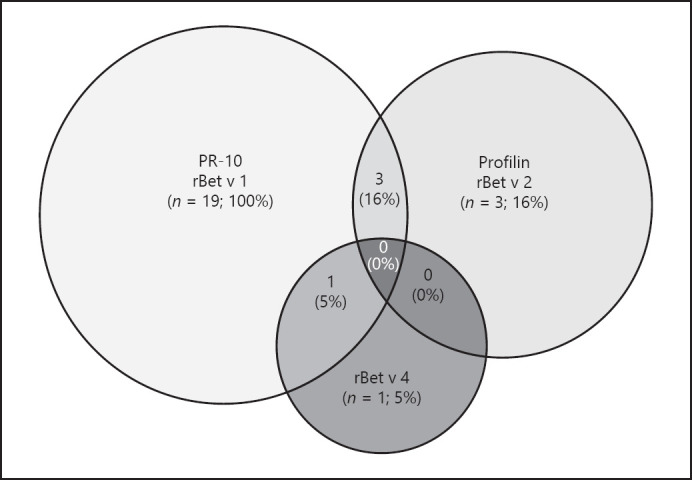
Sensitization rates to birch pollen allergens measured by ImmunoCAP® ISAC: 4 patients showed sIgE to two birch pollen allergens. Three out of nineteen subjects were sensitized to rBet v 1 and rBet v 2, and 1 patient was sensitized to rBet v 1 and rBet v 4.

In consistence with the ImmunoCAP®-FEIA test results, all patients measures by ImmunoCAP® ISAC showed rBet v 1-specific IgE, whereas one responder and two non-responders showed sensitizations to rBet v 2, and in one responder, sIgE antibodies to birch pollen polcalcin rBet v 4 were detected (shown in Fig. 1). In addition to the sensitization to rBet v1, all responders and non-responders showed sIgE to the major allergens of hazel (Cor a 1.0101) and alder (Aln g 1).

Moreover, all patients showed IgE sensitization to at least three birch pollen-related food allergens. In more detail, more than half of the study cohort were IgE-sensitized to major birch pollen-related food allergens of the PR-10 allergen super family: rMal d 1 (100%), rPru p 1 (100%), rCor a 1.0401 (100%), rAra h 8 (95%), rGly m 4 (84%), and rApi g 1 (58%). Sensitization rates to carrot allergen rDau c 1 and kiwi allergen nAct d 8 were considerably lower (37% and 32%, respectively) (shown in Table 2).

Overall, there was no statistically significant difference in sensitization rates to birch pollen-related food and early flowering aeroallergens between responders and non-responders (data not shown). Responders and non-responders did not differ significantly in terms of the degree of sensitizations to rBet v 1 and tested Bet v 1 homolog food allergens measured with the ImmunoCAP® ISAC microarray (shown in Table 3). Likewise, no significant differences regarding the degree of sensitizations could be observed in subjects with a late-type eczematous reaction (*n* = 7) compared to those who did not show a worsening of AD upon DBPCFC (*n* = 12) (*p* > 0.50, Mann-Whitney U-test) (data not shown).

### Correlation of Total IgE and Sensitization Rates Detected with ImmunoCAP® ISAC in AD Patients

**Fig. 2:**
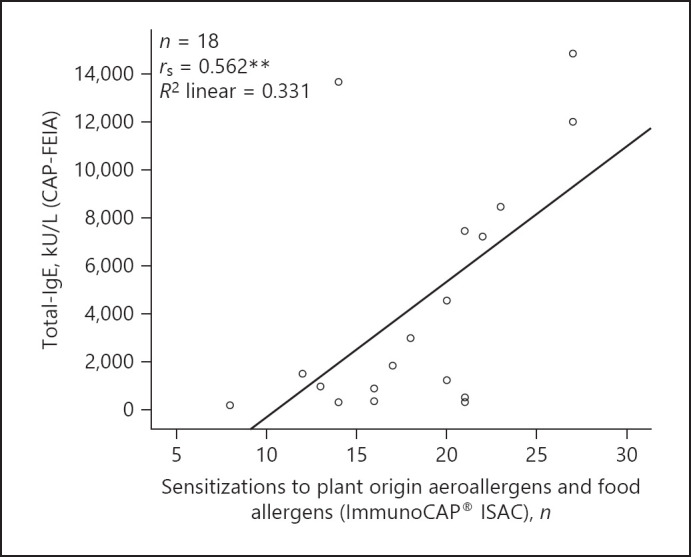
Correlation between total IgE titers detected with ImmunoCAP®-FEIA and number of sensitizations to plant origin allergens in ImmunoCAP® ISAC. *n*, number of subjects; *r*_*s*_, Spearman's rank correlation coefficient; *R*^2^, coefficient of determination. **Highly significant (*p* < 0.001).

Total IgE titers detected with ImmunoCAP®-FEIA and the number of sensitizations to plant origin allergens detected with ImmunoCAP® ISAC were positive and highly significantly correlated (*r*_*s*_ = 0.562, *p* < 0.001) (shown in Fig. 2). No correlation could be observed between disease severity assessed by the SCORAD index and number of sensitizations in ImmunoCAP® ISAC (*r*_s_ = 0.104) (data not shown).

#### Highly Significant Correlations between sIgE Measurements from ImmunoCAP®-FEIA and ImmunoCAP® ISAC

To determine the diagnostic validity of ImmunoCAP® ISAC, sensitization rates to birch pollen allergens and birch pollen-related foods (apple, carrot, celery, and hazelnut) detected with the microarray were compared with the ImmunoCAP®-FEIA singleplex test results (shown in Table 4). There was a strongly positive and highly significant correlation between both test systems in terms of rBet v 1 (*r*_s_ = 0.823) and rBet v 2 (*r*_s_ = 0.983) as well as in case of rBet v 1 measured with ImmunoCAP® ISAC and birch pollen extract measured with ImmunoCAP®-FEIA (*r*_s_ = 0.913). Correlation coefficients of food-specific IgE (apple, carrot, celery, and hazelnut) were lower between both test systems but also statistically significant (shown in Table 4).

## Discussion

In our work, detailed sensitization profiles to plant origin aeroallergens and food allergens could be displayed to investigate the validity of microarray analysis for diagnosis of birch pollen-related food allergy in birch pollen-sensitized AD patients. In accordance to our previously published data [18], AD patients responding to birch pollen food showed a clear trend for a higher prevalence of RCA compared to the non-responders that did not reach the level of statistical significance due to small sample size.

The major cause for cross-reactivity between birch pollen and birch pollen-related food allergens is the major birch pollen allergen Bet v 1 [2]. All birch pollen food-allergic patients identified by DBPCFC (responders) in this study but also all non-responders were sensitized to rBet v 1 when measured by ImmunoCAP® ISAC. This result is consistent with the inclusion criterium of an existing IgE-mediated sensitization to birch pollen extract of at least ≥3.5 kU/L. In accordance to previously published data, the observed sensitization rate to birch pollen profilin rBet v 2 was 16% in our study population [2, 37]. Only 1 patient was additionally IgE-sensitized to rBet v 4 which is established as a minor birch pollen allergen (polcalcin pan allergen) [44]. In conclusion, sensitizations to birch pollen allergens rBet v1, rBet v 2, and rBet v 4 did finally not serve to predict a birch pollen-related food allergy in this study cohort of AD patients with a history of birch pollen-related food allergy.

Furthermore, different sensitization rates to Bet v 1 homolog food allergens could be observed. Sensitization rates to the major pollen allergens from hazel (rCor a 1.0101) and alder (rAln g 1) were 100%. This observation can be explained by the high similarity degree between these allergens belonging to the PR-10 allergen super family and the major birch pollen allergen Bet v 1 [45].

PR-10 homolog allergens from apple (rMal d 1), hazelnut (rCor a 1.0401), peach (rPru p 1), peanut (rAra h 8), and soy (rGly m 4) were recognized by more than 80% of investigated patients, while Bet v 1 homolog allergens from celery, carrot, and kiwi were positive in less than 60% of the patients. Higher sensitization rates to major allergens from apple and hazelnut compared to those from celery and carrot in our study population can be explained by higher similarity degrees between Bet v 1 and Cor a 1.0401 and Mal d 1 (79% and 71%, respectively) compared to Api g 1 and Dau c 1 (61% and 57%, respectively) that determines the degree of IgE-mediated cross-reactivity [46].

Sensitization profiles dominated by proteins from the PR-10 family seen in our study were also published by Röckmann et al. [23] who investigated the pattern of food sensitization in adults with AD in relation to AD severity using multiplexed allergen microarray ImmunoCAP® ISAC. Regarding symptoms of birch pollen-related food allergy in our study cohort, these sensitization profiles correlated with a predominance of mild oropharyngeal symptoms seen in the responders but did not differentiate between responders and non-responders.

We further addressed the question whether responders in our study are characterized by distinct sensitizations other than birch pollen allergens. First, we observed that total IgE titers and numbers of sensitizations in ImmunoCAP® ISAC were highly significantly positively correlated (*r*_s_ = 0.562, *p* < 0.001). These results confirm previous observations from Ott et al. who investigated individual sIgE recognition patterns by microarray analysis also in relation to the disease severity in 20 patients with AD [20]. By contrast to Ott et al. [20], no correlation could be observed between disease severity assessed by the SCORAD index and number of sensitizations detected with microarray analysis in our study cohort.

Furthermore, our data analysis did not reveal any differences in sensitization profiles to birch pollen-related aero- and food allergens assessed with allergen microarray ImmunoCAP® ISAC neither between responders and non-responders nor between those patients who showed worsening of AD after DBPCFC and those without late-type eczematous response. These observations are in line with the results of Wöhrl et al. [47] who focused on RCA to different aeroallergens including birch pollen allergens. There were no differences in sensitization profiles assessed by ImmunoCAP® ISAC to aeroallergens detected with microarray in symptomatic patients with rhinoconjunctival symptoms and asymptomatic patients [47]. Likewise, in a study of Ebo et al. [19], microanalysis was not sufficient to discriminate between birch pollen-allergic subjects with and without a manifested birch pollen-related apple allergy, showing an OAS upon apple ingestion.

We also compared the diagnostic validity of ImmunoCAP® ISAC versus ImmunoCAP®-FEIA. In several studies, strong correlation between the ImmunoCAP® ISAC test and singleplex tests has been demonstrated for different aero- and food allergens [20, 24, 25, 26, 30, 35]. Indeed, our work affirms that there is a highly significant positive correlation between sIgE detection with ImmunoCAP®-FEIA and ImmunoCAP® ISAC in terms of rBet v 1 and rBet v 2 as well as in terms of birch pollen extract (ImmunoCAP®-FEIA) and rBet v 1 (ImmunoCAP® ISAC) (*r*_s_ > 0.8).

Extended analysis further revealed only a lower correlation between sIgE to birch pollen-related food allergen extracts of apple, carrot, celery, and hazelnut detected with ImmunoCAP®-FEIA and sIgE detection to recombinant Mal d 1 from apple, Dau c 1 from carrot, Api g 1 from celery, and Cor a 1.0101 from hazelnut detected with ImmunoCAP® ISAC (*r*_s_ 0.507–0.784) in our study population which was however significant in all cases. The lower correlation can be explained with a confounding by cross-reactive allergen components such as profilins and cross-reactive carbohydrates which are present in plant-origin allergen extracts but not in recombinant allergen components used in ImmunoCAP® ISAC. It may also be speculated that allergens from the PR-10 family are not well represented in allergen extracts used for in vitro diagnosis [37]. In fact, these results are in line with those from Ott et al. [20] who also showed high correlation between both test systems for recombinant allergen components (*r*_s_ 0.81–0.99) but not for allergen extracts measured with ImmunoCAP®-FEIA in comparison to recombinant allergen components detected with ImmunoCAP® ISAC (*r*_s_ 0.72–0.76) [20].

The main limitation of our study that should be considered in the interpretation of the presented results is the small sample size of 19 subjects and its retrospective and monocentric design. Our results refer to a small number of a highly selected patient population of birch pollen-sensitized AD patients and cannot be transferred to general patient populations, especially not to those outside birch-endemic regions. Furthermore, the small number of participants reduces the statistical power to evaluate diagnostic validity of the allergen microarray ImmunoCAP® ISAC. Further prospective multicenter studies are needed to confirm these results in larger patient cohorts from different geographical areas.

In conclusion, our current work confirms that allergen microarray ImmunoCAP® ISAC allows a precise differentiation of the patient's sensitization profile from their response to Bet v 1-homolog aero- and food allergens as well as to other recombinant and native allergen components. However, microarray analysis is not a suitable diagnostic tool to discriminate neither between subjects with a clinical relevant birch pollen-related food allergy nor between those with a late-type eczematous reaction to birch pollen-related foods (apple, carrot, celery, and/or hazelnut) and those who did not show a worsening of AD upon oral food challenge. Therefore, ImmunoCAP® ISAC as a single diagnostic tool is insufficient to diagnose a clinical relevant immediate or late-type birch pollen-related food allergy in patients with AD with a history of birch pollen-related food allergy. Clinical relevance of microarray test results should be evaluated carefully in patient subgroups with clear clinical phenotypes before reaching of therapeutic decisions.

## Statement of Ethics

In all patients, serological tests for allergy detecting sIgE antibodies has been conducted as a routine diagnostic procedure in the Department of Dermatology and Allergy, Hannover Medical School, Germany. Furthermore, all patients had consecutively undergone oral food challenges with birch pollen-related foods as a routine diagnostic procedure in the Department of Dermatology and Allergy, Hannover Medical School, Germany, according to the guidelines on food allergy. Prior to oral food challenges, written informed consent had been obtained from all patients. Corresponding data were analyzed retrospectively. Therefore, an approval of an Ethics Committee was not required.

## Conflict of Interest Statement

Anja Wassmann-Otto has received honoraria for a presentation from Pfizer. Annice Heratizedeh has received consultancy fees from Lilly, Novartis, Pierre Fabre, Sanofi-Genzyme, and Beiersdorf. She has received payment or honoraria for lectures, presentations, speaker's bureaus, manuscript writing, or educational events from AbbVie, LEO Pharma, Novartis, Pierre Fabre, Sanofi-Genzyme, Beiersdorf, Nutricia, Hans Karrer, and Meda as well as travel and attending meeting grants from AbbVie and Janssen. Annice Heratizadeh has participated in an advisory board from Klinge. Additionally, she is a member of the advisory board of the executive committee of the AGNES e.V. (patient education group). Thomas Werfel has received consultancy fees from Sanofi-Regeneron, Lilly, AbbVie, and Novartis. He has received speaker's honorarium from Almriall Hermal and grants from AbbVie, Novartis, LEO Pharma, and Pfizer. He has participated in advisory boards from AbbVie, Pfizer, Lilly, and LEO Pharma. He is a board member of the DGAKI and a board member of allergy guideline of the EAACI.

Katja Wichmann declares that no conflict of interest exists. The authors declare no conflict of interest relating to this article.

## Funding Sources

Thermo Fisher Scientific Inc provided a discount for the use of the ImmunoCAP® ISAC multiplex system for clinical routine.

## Author Contributions

Anja Wassmann-Otto analyzed, interpreted, and discussed the data and wrote the manuscript. Annice Heratizadeh discussed the data and revised the article critically. Katja Wichmann discussed the data and revised the article critically. Thomas Werfel conceived and designed the retrospective analyses, discussed the data, and revised the article critically.

## Data Availability Statement

All data generated or analyzed during this study are included in this article and its supplementary material files. Further inquiries can be directed to the corresponding author.

## Supplementary Material

Supplementary data

## Figures and Tables

**Table 1:** Median sIgE titers to birch pollen allergens and birch pollen-related foods detected by ImmunoCAP^®^-FEIA in responders and non-responders

IgE [kU/L]	Responders (R)	Non-responders (N)	*p* value^1^
Total IgE (*n*_R_ = 11; *n*_N_ = 7)	1,476.00	1,819.00	0.964
sIgE birch (*n*_R_ = 12; *n*_N_ = 7)	61.05	28.6	0.828
Ratio sIgE birch/total IgE (*n*_R_ = 11; *n*_N_ = 7)	0.013	0.016	0.964
sIgE rBet v 1 (*n*_R_ = 9; *n*_N_ = 4)	70.70	76.75	0.634
sIgE rBet v 2 (*n*_R_ = 4; *n*_N_ = 4)	<0.35	<0.35	0.462
sIgE apple (*n*_R_ = 12; *n*_N_ = 7)	3.58	1.66	0.642
sIgE carrot (*n*_R_ = 12; *n*_N_ = 7)	2.88	1.74	0.583
sIgE celery (*n*_R_ = 12; *n*_N_ = 7)	3.70	1.48	0.581
sIgE hazelnut (*n*_R_ = 12; *n*_N_ = 7)	9.88	10.70	0.933

sIgE, specific IgE; *n*_R_, number of responders; *n*_N_, number of non-responders.

1 Mann-Whitney U-test.

**Table 2:** ImmunoCAP^®^ ISAC sensitization profile of responders (*n* = 12) and non-responders (*n* = 7): birch pollen allergens, PR-10-related aeroallergens, and PR-10-related food allergens (imaging referring to [20])

Allergen source	IUIS	Patient ID																				No. pos
	responders												non-responders							
		1	2	3	4	5	6	7	8	9	10	11	12		13	14	15	16	17	18	19	
Kiwi	nAct d 8																					**6**

Celery	rApi g 1																					**11**

Carrot	rDau c 1																					**7**

Apple	rMal d 1																				**19**

Peach	rPru p 1																					**19**

Peanut	rAra h 8																					**18**

Hazelnut	rCor a 1.0401																					**19**

Soybean	rGly m 4																					**16**

No. sensitizations		**6**	**8**	**5**	**5**	**8**	**7**	**7**	**5**	**7**	**8**	**5**	**3**		**7**	**6**	**5**	**6**	**6**	**5**	**6**	

Allergen source	IUIS	Patient ID																			No. pos
		responders												non-responders							
		1	2	3	4	5	6	7	8	9	10	11	12		13	14	15	16	17	18	19	
Birch	rBet v 1																					**19**
	rBet v 2																					**3**
	rBet v 4																					**1**

Alder	rAln g 1																					**19**

Hazel pollen	rCor a 1.0101																					**19**

No. sensitizations		**3**	**3**	**3**	**3**	**3**	**3**	**3**	**3**	**4**	**4**	**3**	**3**		**4**	**3**	**3**	**3**	**3**	**3**	**4**	

White, undetectable (<0.3 ISU); light gray, low (0.3–0.9 ISU); medium gray, moderate/high (1–14.9 ISU); dark gray, very high (≥15 ISU). ISU, immuno solid-phase allergen chip standardized units; IUIS, International Union of Immunological Societies.

**Table 3:** Comparison of ImmunoCAP^®^ ISAC test results of responders and non-responders: PR-10 proteins (medians)

IgE (ISU)	Responders (*n* = 12)	Non-responders (*n* = 7)	*p* value*
rBet v 1	49.5 (95% CI: 19.0–80.5)	58.0 (95% CI: 24.0–60.0)	0.833
rAln g 1	10.3 (95% CI: 3.6–35.0)	9.1 (95% CI: 3.5–18.0)	0.704
rCor a 1.0101	12.2 (95% CI: 5.6–38.0)	8.7 (95% CI: 6.4–27.0)	0.612
rMal d 1	15.0 (95% CI: 3.7–34.5)	6.2 (95% CI: 4.3–14.0)	0.331
rDau c 1	<0.3 (95% CI <0.3–0.7)	<0.3 (95% CI <0.3–1.4)	0.807
rApi g 1	0.5 (95% CI <0.3–4.1)	1.0 (95% CI: 0.3–3.2)	0.603
rCor a 1.0401	9.1 (95% CI: 3.3–24.0)	16.0 (95% CI: 4.3–22.0)	0.735
rGly m 4	2.6 (95% CI: 1.3–10.5)	1.3 (95% CI <0.3–1.9)	0.069
rAra h 8	7.5 (95% CI 4.2–22.5)	8.1 (95% CI 0.4–13.0)	0.966
rPru p 1	12.5 (95% CI 3.0–23.0)	6.9 (95% CI 3.6–17.0)	0.554
nAcd d 8	<0.3 (95% CI <0.3–0.6)	<0.3 (95% CI <0.3–<0.3)	0.219

ISU, immuno solid-phase allergen chip standardized units.

* Mann-Whitney U-test.

**Table 4:** Correlation between sensitization rates measured with ImmunoCAP^®^-FEIA and ImmunoCAP^®^ ISAC

Allergen source	ISAC microarray (allergen component)	FEIA (allergen component/extract)	*r*_s_
Birch	rBet v 1	Birch (t3)	0.913**
	rBet v 1	rBet v 1 (t215)	0.823**
	rBet v 2	rBet v 2 (t216)	0.983**
Apple	rMal d 1	Apple (f49)	0.784**
Carrot	rDau c 1	Carrot (f31)	0.507*
Celery	rApi g 1	Celery (f85)	0.586**
Hazelnut	rCor a 1.0401	Hazelnut (f17)	0.519**

*r*_s_, spearman's rank correlation coefficient.

* Significant (*p* < 0.05).

** Highly significant (*p* < 0.001).

## Notes

Edited by: O. Palomares, Madrid.
