# Supplementary material for: Microarray Analysis Confirms ImmunoCAP-Fluorescence Enzyme Immunoassay Results on Specific IgE in Patients with Atopic Dermatitis and Suspected Birch Pollen-Related Food Allergy
Source: Int Arch Allergy Immunol. 2022 Apr 4;183(8):814–23. doi: 10.1159/000522525 (PMC9533431; doi:10.1159/000522525)
Supplement: Supplementary file 1 — Supplementary data [file iaa-0183-0814-s01.docx]

**Supplement 1: (a)** ImmunoCAP^®^ ISAC sensitization profile of responders (n=12)

and non-responders (n=7): Plant origin aeroallergens (except for birch pollen- and PR-10-related allergens) and latex allergens (imaging referring to [20])

| **Allerge source** | **IUIS** | **Patient ID** | | | | | | | | | | | | | | | | | | | **No. pos.** |
| --- | --- | --- | --- | --- | --- | --- | --- | --- | --- | --- | --- | --- | --- | --- | --- | --- | --- | --- | --- | --- | --- |
|  |  | **Responders** | | | | | | | | | | | | **Non-Responders** | | | | | | |  |
|  |  | **1** | **2** | **3** | **4** | **5** | **6** | **7** | **8** | **9** | **10** | **11** | **12** | **13** | **14** | **15** | **16** | **17** | **18** | **19** |  |
| **Bermuda gras** | rCyn d 1 |  |  |  |  |  |  |  |  |  |  |  |  |  |  |  |  |  |  |  | **17** |
| **Timothy gras** | rPhl p 1 |  |  |  |  |  |  |  |  |  |  |  |  |  |  |  |  |  |  |  | **16** |
|  | rPhl p 2 |  |  |  |  |  |  |  |  |  |  |  |  |  |  |  |  |  |  |  | **8** |
|  | nPhl p 4 |  |  |  |  |  |  |  |  |  |  |  |  |  |  |  |  |  |  |  | **13** |
|  | rPhl p 5 |  |  |  |  |  |  |  |  |  |  |  |  |  |  |  |  |  |  |  | **14** |
|  | rPhl p 6 |  |  |  |  |  |  |  |  |  |  |  |  |  |  |  |  |  |  |  | **11** |
|  | rPhl p 7 |  |  |  |  |  |  |  |  |  |  |  |  |  |  |  |  |  |  |  | **1** |
|  | rPhl p 11 |  |  |  |  |  |  |  |  |  |  |  |  |  |  |  |  |  |  |  | **8** |
|  | rPhl p 12 |  |  |  |  |  |  |  |  |  |  |  |  |  |  |  |  |  |  |  | **4** |
| **Japanese ceder** | nCry j 1 |  |  |  |  |  |  |  |  |  |  |  |  |  |  |  |  |  |  |  | **5** |
| **Cypress** | nCup a 1 |  |  |  |  |  |  |  |  |  |  |  |  |  |  |  |  |  |  |  | **4** |
| **Olive** | nOle e 1 |  |  |  |  |  |  |  |  |  |  |  |  |  |  |  |  |  |  |  | **5** |
|  | nOle e 2 |  |  |  |  |  |  |  |  |  |  |  |  |  |  |  |  |  |  |  | **3** |
| **Plane tree** | rPla a 1 |  |  |  |  |  |  |  |  |  |  |  |  |  |  |  |  |  |  |  | **0** |
|  | rPla a 2 |  |  |  |  |  |  |  |  |  |  |  |  |  |  |  |  |  |  |  | **8** |
| **Ragweed** | rAmb a 1 |  |  |  |  |  |  |  |  |  |  |  |  |  |  |  |  |  |  |  | **0** |
| **Mugwort** | rArt v 1 |  |  |  |  |  |  |  |  |  |  |  |  |  |  |  |  |  |  |  | **6** |
|  | rArt v 3 |  |  |  |  |  |  |  |  |  |  |  |  |  |  |  |  |  |  |  | **5** |
| **Wall pellitory** | rPar j 2 |  |  |  |  |  |  |  |  |  |  |  |  |  |  |  |  |  |  |  | **3** |
| **Saltwort** | nSal k 1 |  |  |  |  |  |  |  |  |  |  |  |  |  |  |  |  |  |  |  | **2** |
| **Annual mercury** | rMer a 1 |  |  |  |  |  |  |  |  |  |  |  |  |  |  |  |  |  |  |  | **3** |
| **Latex** | rHev b 1 |  |  |  |  |  |  |  |  |  |  |  |  |  |  |  |  |  |  |  | **1** |
|  | rHev b 3 |  |  |  |  |  |  |  |  |  |  |  |  |  |  |  |  |  |  |  | **1** |
|  | rHev b 5 |  |  |  |  |  |  |  |  |  |  |  |  |  |  |  |  |  |  |  | **1** |
|  | rHev b 6 |  |  |  |  |  |  |  |  |  |  |  |  |  |  |  |  |  |  |  | **3** |
|  | rHev b 8 |  |  |  |  |  |  |  |  |  |  |  |  |  |  |  |  |  |  |  | **3** |
| **No. sensitizations** | | **11** | **5** | **5** | **4** | **13** | **6** | **11** | **9** | **14** | **9** | **10** | **0** | **10** | **2** | **4** | **7** | **7** | **9** | **6** |  |

IUIS: International Union of Immunological Societies

White: Undetectable (<0,3 ISU), striped: Low (0,3-0,9 ISU), grey: Moderate/high (1-14,9 ISU), black: Very high (≥15 ISU) (ISU: ISAC Standardized Units)

**Supplement 2: (b)** ImmunoCAP^®^ ISAC sensitization profile of responders (n=12)

and non-responders (n=7): Plant origin food allergens (except for PR-10-related allergens) (imaging referring to [20])

| **Allergen source** | **IUIS** | **Patient ID** | | | | | | | | | | | | | | | | | | | **No. pos.** |
| --- | --- | --- | --- | --- | --- | --- | --- | --- | --- | --- | --- | --- | --- | --- | --- | --- | --- | --- | --- | --- | --- |
|  |  | **Responders** | | | | | | | | | | | | **Non-Responders** | | | | | | |  |
|  |  | **1** | **2** | **3** | **4** | **5** | **6** | **7** | **8** | **9** | **10** | **11** | **12** | **13** | **14** | **15** | **16** | **17** | **18** | **19** |  |
| **Kiwi** | nAct d 1 |  |  |  |  |  |  |  |  |  |  |  |  |  |  |  |  |  |  |  | **4** |
|  | nAct d 2 |  |  |  |  |  |  |  |  |  |  |  |  |  |  |  |  |  |  |  | **5** |
|  | nAct d 5 |  |  |  |  |  |  |  |  |  |  |  |  |  |  |  |  |  |  |  | **0** |
| **Peach** | nPru p 3 |  |  |  |  |  |  |  |  |  |  |  |  |  |  |  |  |  |  |  | **3** |
| **Cashew nut** | rAna o 2 |  |  |  |  |  |  |  |  |  |  |  |  |  |  |  |  |  |  |  | **0** |
| **Peanut** | nAra h 1 |  |  |  |  |  |  |  |  |  |  |  |  |  |  |  |  |  |  |  | **1** |
|  | nAra h 2 |  |  |  |  |  |  |  |  |  |  |  |  |  |  |  |  |  |  |  | **1** |
|  | nAra h 3 |  |  |  |  |  |  |  |  |  |  |  |  |  |  |  |  |  |  |  | **0** |
| **Brazil nut** | rBer e 1 |  |  |  |  |  |  |  |  |  |  |  |  |  |  |  |  |  |  |  | **0** |
| **Hazelnut** | rCor a 8 |  |  |  |  |  |  |  |  |  |  |  |  |  |  |  |  |  |  |  | **3** |
|  | nCor a 9 |  |  |  |  |  |  |  |  |  |  |  |  |  |  |  |  |  |  |  | **0** |
| **Soybean** | nGly m 5 |  |  |  |  |  |  |  |  |  |  |  |  |  |  |  |  |  |  |  | **0** |
|  | nGly m 6 |  |  |  |  |  |  |  |  |  |  |  |  |  |  |  |  |  |  |  | **0** |
| **Sesame seed** | nSes i 1 |  |  |  |  |  |  |  |  |  |  |  |  |  |  |  |  |  |  |  | **0** |
| **Wheat** | nTri a 18 |  |  |  |  |  |  |  |  |  |  |  |  |  |  |  |  |  |  |  | **1** |
|  | nTri a Gliadin |  |  |  |  |  |  |  |  |  |  |  |  |  |  |  |  |  |  |  | **3** |
|  | rTri a 19.0101 |  |  |  |  |  |  |  |  |  |  |  |  |  |  |  |  |  |  |  | **0** |
|  | nTri a aA_TI |  |  |  |  |  |  |  |  |  |  |  |  |  |  |  |  |  |  |  | **0** |
| **Bromelain** | nAna c 2 |  |  |  |  |  |  |  |  |  |  |  |  |  |  |  |  |  |  |  | **6** |
| **No. sensitizations** | | **2** | **0** | **1** | **0** | **3** | **1** | **2** | **1** | **2** | **0** | **3** | **2** | **0** | **2** | **2** | **0** | **2** | **0** | **4** |  |

IUIS: International Union of Immunological Societies

nTri aA_TI: nTri a Alpha-Amylase/Trypsin-Inhibitor

White: Undetectable (<0,3 ISU), striped: Low (0,3-0,9 ISU), grey: Moderate/high (1-14,9 ISU), black: Very high (≥15 ISU) (ISU: ISAC Standardized Units)
